# Homocysteine and methylmalonic acid in Phenylketonuria patients

**DOI:** 10.1590/1678-4685-GMB-2023-0103

**Published:** 2024-04-05

**Authors:** Giovana Regina Weber Hoss, Fernanda Sperb-Ludwig, Tássia Tonon, Soraia Poloni, Sidney Behringer, Henk J. Blom, François Maillot, Ida Vanessa Doederlein Schwartz

**Affiliations:** 1 Hospital de Clínicas de Porto Alegre, Laboratório BRAIN, Porto Alegre, RS, Brazil.; 2 Universidade Federal do Rio Grande do Sul (UFRGS), Programa de Pós-Graduação em Genética e Biologia Molecular, Porto Alegre, RS, Brazil.; 3 University Medical Centre, Laboratory of Clinical Biochemistry and Metabolism, Freiburg, Germany.; 4 Hospital de Clínicas de Porto Alegre, Serviço de Genética Médica, Porto Alegre, RS, Brazil.; 5 Erasmus Universiteit Rotterdam, Laboratory of Clinical Genetics, The Netherlands.; 6 University Hospital of Tours, Department of Internal Medicine, Tours, France.; 7 UMR INSERM 1253, Tours, France.; 8 Reference Center for Inherited Metabolic Diseases, Tours, France.

**Keywords:** Phenylketonuria, Homocysteine, Methylmalonic Acid, Vitamin B12

## Abstract

Hyperhomocysteinemia and vitamin B12 deficiency have been reported in patients with phenylketonuria. In this study, total homocysteine (tHcy) and methylmalonic acid (MMA) levels were analyzed in samples from 25 phenylketonuria (PKU) patients. Comparisons were made between pre- and post-treatment values (n= 3); on treatment values, between periods with high and normal/low phenylalanine (Phe) levels (n= 20); and in women before, during and after pregnancy (n= 3). THcy levels decreased after treating PKU with metabolic formula (p=0.014). Except for a pregnant woman before pregnancy, none of the patients had tHcy values above the normal range. In fact, tHcy was < 5 μmol/L in 34% of the samples. We observed a decrease in Phe, tHcy, and tyrosine levels during pregnancy. MMA levels did not differ significantly, with values remaining in the normal range. These data indicate that there was no B12 deficiency in patients who adhere to the diet. In conclusion, in PKU patients treated with metabolic formula, tHcy is frequently not elevated, remaining even in the lower normal range in some patients. Thus, clinical follow-up and adherence to dietary treatment are crucial to prevent B12 deficiency.

## Introduction

Phenylketonuria (PKU) is an autosomal recessive metabolic disorder characterized by phenylalanine hydroxylase (PAH) deficiency, in which the essential amino acid phenylalanine (Phe) cannot be converted to tyrosine (Tyr). Mutations in the *PAH* gene lead to accumulation of toxic metabolites (Dobbelaere et al., 2003; Blau et al., 2010; Flydal and Martinez, 2013).

PKU treatment is mainly dietary and consists of Phe restriction by limiting natural protein intake, typically to < 10 g/day, in combination with a Phe-free amino acid mixture enriched with vitamins and trace minerals (metabolic formula) (MacDonald et al., 2011). The metabolic formula supplies 50%-85% of total daily protein requirements (Singh et al., 2014). Early initiation (i.e., shortly after birth) of a Phe-restricted diet for PKU patients avoids neuropsychological complications (Blau et al., 2010). Nevertheless, nutritional deficiencies, such as impaired growth, reduced bone mineral density, and micronutrient deficiencies have been described (Dobbelaere et al., 2003; Blau *et al*., 2010). Oral treatment with the cofactor of PAH, BH4, can be indicated for responsive patients.

Vitamin B12 is mainly present in animal-based foods, so patients on protein-restricted diets, such as PKU patients, are at risk of B12 deficiency (Vugteveen et al., 2011; Procházková et al., 2015). Although metabolic formula includes B12 supplement, many patients do not take the formula as prescribed due to its low palatability. Studies of PKU patients on dietary treatment have shown conflicting results: some report higher levels of B12 than healthy controls (Huemer et al., 2008; Stølen et al., 2014; Andrade et al., 2017). Kose *et al*. observed that PKU patients had higher levels of B12, and that B12 deficiency occurred in 15% of PKU patients vs 30% of healthy controls (Kose and Arslan, 2018). However, comparing PKU patients who were adherent and non-adherent to the diet, Schulpis et al. (2002) found higher levels of homocysteine (Hcy) in adherent patients and decreased levels of B12, B6, and folate in non-adherent ones (Schulpis *et al*., 2002). We did not find differences between periods patients 

Vitamin B12 is essential for hematological and neurological processes and serves as a cofactor in 2 enzymatic reactions: 1) conversion of methylmalonyl-CoA to succinyl-CoA; 2) remethylation of homocysteine to methionine (Met). In vitamin B12 deficiency, methylmalonyl-CoA is converted to methylmalonic acid (MMA) (Prick et al., 2012; Gaiday et al., 2018). Consequently, in B12 deficiency, total Hcy (tHcy) and methylmalonic acid (MMA) increase in plasma and, thus, are important functional biomarkers of vitamin B12 status (Vugteveen et al., 2011). 

Women with PKU and poor Phe control during pregnancy are at high risk of having a child with intellectual disability, congenital heart defects, intrauterine growth retardation, and other defects, since Phe is teratogenic. Therefore, Phe levels < 360 μmol/L prior to conception and throughout pregnancy are recommended (Rouse and Azen, 2004; Prick et al., 2012). In general, pregnant women with low levels of vitamin B12 and folate and/or high Hcy levels are at higher risk of pregnancy complications, such as neural tube defects, recurrent pregnancy loss, preeclampsia, prematurity, and poor birth outcomes (Nelen, 2001; Gaiday et al., 2018). Plasma tHcy is usually lower during the first two trimesters of pregnancy and returns to preconception concentrations during late pregnancy (Murphy et al., 2004). Murphy *et al*. (2002) concluded that this variation is not explained by pregnancy factors such as hemodilution or folic acid supplementation, but it may be related to hormone levels.

Due these conflicting B12 results, we studied the levels of MMA, tHcy, Met, and other aminoacids in PKU patients: 1) pre- and post- dietary treatment, 2) on treatment, at points of high (≥360 μmol/L) and normal or low Phe levels (< 360 μmol/L), and 3) in women with PKU who became pregnant (before, during, and after pregnancy).

## Material and Methods

This cross-sectional study was conducted at the Hospital de Clínicas de Porto Alegre (HCPA), Brazil and was approved by the HCPA ethics committee (n. 2017-0273). All participants or their caretakers provided written informed consent prior to inclusion.

### Patients

A total of 25 patients with PKU were included (22 non-pregnant and 3 pregnant). The patients had a biochemical and/or genetic diagnosis of PKU. In all cases, the diagnosis of BH4 metabolism diseases was excluded biochemically through analyzes of biopterin/pterins and DHPR activity. All patients had elevated Phe levels at diagnosis (equal to or greater than 240 micromol/L) with normal or reduced tyrosine levels. All patients were diagnosed by neonatal screening, except for two who were diagnosed after an investigation for intellectual disability and are under clinical follow-up at HCPA. 

Samples from 20/22 patients (male: 11) were compared in relation to the periods with high Phe and non-high Phe levels. More than one sample was analyzed for all patients, including at least 1 period with a high Phe level and one with a normal or low Phe level. Samples from 3/22 patients were analyzed before and at treatment, and samples were collected before, during, and after pregnancy in 3 other patients.

A structured form was used to gather information about diagnosis, treatment strategies, treatment adherence, metabolic control, and current health condition from the medical record. We correlated the values of amino acids and analytes related to tHcy with Phe levels and dietary treatment adherence. Phe levels ≥ 360 µmol/L were considered indicative of poor metabolic control. 

### Biochemical analysis

Blood samples were taken after a 12 h overnight fast. Immediately after collection, samples were centrifuged for 20 min at 3000 × g and plasma was isolated and stored at -80 °C for further analysis. Plasma tHcy, cysteine (Cys), and Met were measured by liquid chromatography electrospray tandem mass spectrometry (LC-MS/MS) following a protocol adapted from Rafii et al. (2007), Persichilli et al. (2010), and Bártl et al. (2014). For quantification, stable isotope-labeled standards were added to the samples. Dithiothreitol was used to reduce disulfide bonds. Methanol was then added to the mixture to precipitate the proteins. After centrifugation (10,000 × g), the supernatant was evaporated, butylated, and then injected into the LC-MS/MS system (Waters Quattro Premier XE, Waters Corp., Manchester, UK). 

Plasma MMA was determined using the LC-MS-MS method described by Blom, van Rooij, and Hogeveen (2007). After deproteinization by ultra-filtration, an acidified aliquot of the eluate was injected into the high-performance liquid chromatography system to separate MMA and succinic acid, and MMA was then analyzed by LC-MS/MS. 

Phe, Tyr, leucine (Leu), isoleucine (Ile), and valine (Val) levels were determined by LC-MS/MS using the multiple reaction monitoring mode (Chace et al., 1997). Since Met, Leu, Ile, and Val are essential amino acids only acquired through diet, these amino acids were used to evaluate patient protein intake.

Plasma vitamin B12 levels were obtained retrospectively through the review of clinical files. It was measured by electrochemiluminescence using an Elecsys 2010 analyzer (Roche Diagnostics GmbH, Mannheim, Germany).

Hormones levels were not measured in the pregnant women in the present study.

### Statistical analysis

Statistical analysis was performed using IBM SPSS Statistics 23 (IBM, Armonk, NY, USA). Normally distributed continuous variables were expressed as mean and SD, while asymmetrically distributed variables were expressed as median and IQR (P25-P75). Differences between groups were determined using Student’s *t*-test. Asymmetrically distributed variables were evaluated with the Mann-Whitney U test. Associations were determined using Spearman’s correlation coefficient. Analyses before, during, and after pregnancy were compared using mixed-model ANOVA. All tests were 2-tailed, with p < 0.05 considered significant.

## Results

The results are summarized in Table 1 and in the Supplementary Tables. All treated patients were on a low-Phe diet, received metabolic formula, and no patient was taking BH4.

**Table 1 -:** Individual phenylalanine, methionine, B12, homocysteine and methylmalonic acid levels in patients at a moment of high Phe (hPhe) or non high Phe (nhPhe).

Metabolite	Period	P1 F)	P2 (M)	P3 (M)	P4 (F)	P5 (F)	P6 (F)	P7* (M)	P8 (F)	P9 (M)	P10 (M)	P11 (F)	P12* (F)	P13 (F)	P14 (M)	P15 (F)	P16 (M)	P17 (M)	P18 (M)	P19 (M)	P20 (M)	Mean +/-SD or Median (IQ25-75)	P
Age (yr)	nhPhe	4.5	1.7	3.5	8.0	0.1	4.3	35.5	5.25	0.3	1.0	20	60.7	6.25	4.25	3.8	15.1	4.4	15.3	2.0	8.0	10.2 (2.4-13.3)	NS
	hPhe	3.5	2.6	5.0	7.8	0.25	3.15	35.8	5.15	1.75	1.1	18	59.6	6.1	4.6	2.3	16.1	2.8	14.5	1.75	9.5	10.0 (2.4-13.2)	
Phe µmol/L	nhPhe	24.2	26.8	43.8	81.6	84.4	98.6	105	113	167	224	280	289	317	327	329	333	333	343	358	359	284 (99.6-333)	<0.001
	hPhe	556	371	671	413	1180	396	447	512	483	537	521	597	565	492	789	637	544	464	456	723	502 (449 - 585)	
Met µmol/L	nhPhe	63.9	18.2	30.1	76.2	32.9	42.9	23.3	27.1	51.1	61.1	21.4	122	20.8	26.4	31.1	29.2	53.8	24.5	18.9	39.2	30.59 (23.6-53.1)	NS
	hPhe	41.3	22.4	36.4	38.6	29.5	31.5	16.5	24.1	28.8	18.7	23.6	24.5	25.0	46.5	41.4	42.7	17.1	24.2	18.5	73.6	26.8 (22.7 - 40.6)	
Vit B12 pg/mL	nhPhe	-	649	1431	-	-	1233	760	524**	-	965	292	1084	720	836	846	-	-	306	-	1036	836 (586-1060)	NS
	hPhe	1480	1199	-	1595	-	-	-	-	933	-	326	-	-	-	780	457	1060	-	660	-	933 (558-1339)	
tHcy µmol/L	nhPhe	10.5	2.86	7.42	10.0	3.92	3.12	6.80	6.34	4.75	6.24	10.2	5.28	7.26	4.71	5.91	10.0	8.23	7.52	3.94	6.40	6.57 (2.35)	NS
	hPhe	7.28	1.77	3.89	12.5	5.05	2.43	6.05	4.45	5.36	4.92	10.9	4.88	9.37	5.00	6.76	8.37	4.89	11.1	3.76	5.93	6.23 (2.89)	
MMA µmol/L	nhPhe	0.21	0.12	0.17	0.30	0.42	0.13	0.11	0.13	0.44	0.15	0.27	0.22	0.16	0.15	0.28	0.18	0.22	0.34	0.16	0.25	0.18 (0.15-0.28)	NS
	hPhe	0.18	0.11	0.14	0.32	0.18	0.12	0.10	0.10	0.79	0.14	0.32	0.20	0.21	0.20	0.21	0.16	0.20	0.31	0.18	0.22	0.20 (0.14-0.24)	

hPhe: high Phe, nhPhe: non high Phe, * Patients diagnosed after clinical presentation of mental retardation; ** Patient supplemented with polyvitamin; - Data not available. Reference values: Phe: <360 μmol/L; Met: 16-34 μmol/L; tHcy: 5-15μmol/L; Vit B12: >200pg/mL; MMA: 0.5 µmol/L.

### Pre- and post-treatment comparisons (n = 3, Table S1)

**Figure 1 -:**
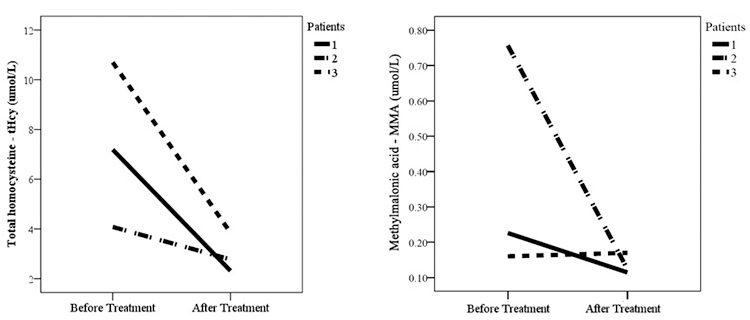
Total homocysteine and methylmalonic acid in PKU patients before and after treatment.

Pre- and post-treatment between-group comparisons indicated significant differences according to the age at which the samples were collected (pretreatment: 2 [0-2] and post-treatment: 27.5 [20.7-41.2] months; p = 0.024). Plasma tHcy levels were higher before (7.32 [SD, 3.31]) than after treatment (2.98 [SD, 0.81]) (p = 0.014). No differences were found in MMA (Figure 1), Met, Tyr, Leu, Ile, or Val. 

### On treatment - high and normal or low Phe comparisons (n = 20; Table 1)

Three patients presented very low Phe levels (pt. 1, 2 and 3). The vitamin B12 levels were in the normal range for all patients, and one patient received multivitamin supplements during the study period. No patients had above-normal tHcy values. In 15 of the 43 samples, the tHcy values were below the reference range (5 μmol/L), but there was no significant difference between timepoints with high or normal/low Phe levels. There were no other significant differences in the evaluated parameters between groups. 

There was a positive correlation between tHcy and MMA r_s_ = 0.486 (p = 0.001), between tHcy and Cys r_s_ = 0.528 (p < 0.001), and between tHcy and age r_s_ = 0.435 (p = 0.004). Met correlated positively with Tyr r_s_ = 0.535 (p < 0.001), Leu r_s_ = 0.707 (p < 0.001), Ile r_s_ = 0.730 (p < 0.001), and Val r_s_ = 0.542 (p < 0.001) (Figure 1). 

### Analysis in pregnant women (n = 3, Table S2)

Analyses in pregnant women showed significant differences in tHcy values (p < 0.001) before and during pregnancy, and before and after childbirth. Only one woman presented high tHcy levels before pregnancy. Tyr levels also differed significantly (p = 0.001), with higher levels in samples collected before pregnancy (79.3 [SD 17.4]) than during pregnancy (43.5 [SD 18.8]), and after childbirth (31.3 [SD 18.7]). Phe levels were significantly lower (p < 0.001) during pregnancy (342.6 [SD 293.1]), with no significant difference in values before (1241.2 [SD 409.8]) and after pregnancy (879.4 [SD 343.2]). No significant differences were found in MMA, Cys, Met, Leu, Ile, or Val among these periods. MMA levels did not differ significantly among the periods. A few samples, about 10%, presented slightly high MMA levels, but the vast majority of samples were within the normal range. There were no significant differences between gestational trimesters. 

## Discussion

We found lower tHcy levels after PKU treatment with low-Phe diet and metabolic formula. No PKU patient besides one woman in the pre-pregancy period had tHcy values above the normal range, indicating no overt deficiencies of folate or B12 among treated patients. This is likely because PKU formulas are enriched with B12 and folate, as shown by Kose and Arslan. (2018). Enrichment may also explain why 34% of PKU patients had tHcy values < 5 μmol/L. 

Vugteveen et al. (2011) analyzed 75 patients with PKU who were receiving treatment, 12 of whom had increased MMA and/or Hcy levels indicative of functional vitamin B12 deficiency. There was no consistent relationship between metabolic control and MMA and Hcy levels, although there were significant correlations between serum vitamin B12 and Hcy, MMA, and metabolic control. Using a combined calculation of vitamin B12, Akış et al. (2020) found lower vitamin B12 in 24.5% of PKU patients and 13.3% of controls, but this difference was not significant.

Like our study, Stølen et al. (2014) found that plasma Hcy levels were below the reference range in 68% of 34 PKU children on dietary treatment. Moreover, plasma levels of folate and vitamin B12 were above the upper reference level in 91% and 53% of the children, respectively. Nevertheless, Huemer et al. (2012) found lower plasma Hcy in 16 children and adolescents with treated PKU compared to age-matched controls, and no difference was found in folate levels. However, another study by this group found no difference in Hcy levels in treated PKU patients (age: 4-20 years) and controls, although folate and vitamin B12 levels were higher in the patient group. 

Karam et al. (2015) found no difference in Hcy levels among 9 patients with PKU (8 children and 1 adult) and a control group (30 healthy subjects, mean age: 12.1 years). Schulpis et al. (2002) compared adherent and non-adherent PKU patients and healthy controls, concluding that these patients had low levels of vitamin B6, vitamin B12, and folate, resulting in moderate hyperhomocysteinemia. Our patients, however, did not differ regarding the values of the metabolites analysed and the periods with high or non-high Phe. The fact that we had the patients showing very low Phe levels can biased the analyses.

By analyzing women before, during, and after pregnancy, we observed a decrease in Phe, tHcy, and Tyr levels during pregnancy. THcy levels were especially high in our PKU patients before pregnancy (mean 22 μmol/L). During pregnancy, tHcy levels were within normal range, similar to those described in healthy pregnant women (Murphy et al., 2002; 2004; Gaiday et al., 2018). 

MMA levels did not differ significantly, with values within the normal range. Akış et al. (2020) found higher MMA concentrations in a PKU group than controls. MMA concentrations were high in 56.5% of the patients and 26.7% of the controls with normal vitamin B12 levels. 

Chowdhury et al. (2011) reported that pregnant women whose child had congenital heart defects had higher levels of Hcy and s-adenosylhomocysteine and lower levels of Met and s-adenosylmethionine. These differences were accompanied by more DNA hypomethylation in mothers than controls. 

The current study was limited by the number and age variability of the patient sample, in addition to the fact that holotranscobalamin and folate could not be measured.

In conclusion, plasma tHcy is not elevated in PKU patients treated with metabolic formula, and in some patients it is even below reference range, so clinical follow-up and adherence to dietary treatment are very important. Plasma amino acids, tHcy, and MMA should be evaluated to detect B12 deficiency in those patients, especially prior to conception to minimize risk to the fetus.

## Supplementary material

The following online material is available for this article:

Table S1 - Individual phenylalanine, methionine, homocysteine, methylmalonic acid, tyrosine, leucine, isoleucine and valine levels in patients before and after treatment.

Table S2 - Individual phenylalanine, methionine, B12, homocysteine and methylmalonic acid levels in patients before, after and pregnancy.
