# Supplementary material for: Homocysteine and methylmalonic acid in Phenylketonuria patients
Source: Genet Mol Biol. 2024 Apr 5;46(3 Suppl 1):e20230103. doi: 10.1590/1678-4685-GMB-2023-0103 (PMC11000623; doi:10.1590/1678-4685-GMB-2023-0103)
Supplement: Table S1 - [file 1415-4757-GMB-46-3-s1-e20230103-s1.pdf]

Genetics and Molecular Biology
https://doi.org/10.1590/1678-4685-GMB-2023-0103

Supplementary Material to “Homocysteine and methylmalonic acid in Phenylketonuria patients”

Table S1 - Individual phenylalanine, methionine, homocysteine, methylmalonic acid, tyrosine, leucine, isoleucine and valine levels in patients before and after treatment.

**Metabolite**	**PT1 (M)**		**PT2 (F)**		**PT3 (M)**		**Mean +/-SD or Median (IQ25-75)**		**P**
	Pre-treatment (n=1)	At-treatment (n=2)	Pre-treatment (n=1)	At-treatment (n=2)	Pre-treatment (n=1)	At-treatment (n=2)	Pre-treatment (n=3)	At-treatment (n=6)	
**Age (months)**	4	25.5	0	44.5	2	22.5	2 (0-2)	27.5 (20.7–41.2)	**0.024**
**Phe (µmol/L)**	604	199	1566	247	689	408	604 (265-604)	366 (80-411)	0.262
**Met (µmol/L)**	15.2	20.3	22.4	37.2	50.9	18.7	29.5 (18.8)	25.4 (9.9)	0.671
**tHcy (µmol/L)**	7.19	2.31	4.08	2.77	10.7	3.85	7.3 (3.3)	2.9 (0.8)	**0.014**
**MMA (µmol/L)**	0.23	0.11	0.76	0.12	0.16	0.17	0.22 (0.16-0.22)	0.27(0.11-0.16)	0.095
**Tyr (µmol/L)**	29.9	69.4	60.9	82.9	61.5	56.5	50.7 (18.1)	69.6 (19.9)	0.212
**Leu (µmol/L)**	56.1	79.8	164.0	233.6	131.8	120.7	117.3 (55.3)	144.7 (73.8)	0.592
**Ile (µmol/L)**	35.4	42.2	96.5	131.3	71.6	64.2	67.8 (30.7)	79.3(44.3)	0.704
**Val (µmol/L)**	99.2	95.5	271.4	388.8	240.7	253.1	203.7 (91.8)	245.8 (139.8)	0.656

Reference values: Phe: <360 µmol/L; Met: 13-45 µmol/L (>1 month), 12-50 µmol/L (1-23 months), 14-37 µmol/L (2-17yo); tHcy: 5-15µmol/L; MMA: 0.1-0.5 µmol/L; Tyr:33-160 µmol/L (>1 month), 24-125 µmol/L (1-23 months), 31-108 µmol/L (2-17yo); Leu: 23-172 µmol/L (>1 month), 43-181 µmol/L (1-24 months), 65-179 µmol/L (2-17yo); Ile: 12-92 µmol/L (>1 month), 10-109 µmol/L (1-24 months), 33-97 µmol/L (2-17yo); Val: 57-250 µmol/L (>1 month), 84-354 µmol/L (1-24 months), 130-307 µmol/L (2-17yo).
