# Supplementary material for: Homocysteine and methylmalonic acid in Phenylketonuria patients
Source: Genet Mol Biol. 2024 Apr 5;46(3 Suppl 1):e20230103. doi: 10.1590/1678-4685-GMB-2023-0103 (PMC11000623; doi:10.1590/1678-4685-GMB-2023-0103)
Supplement: Table S2 - [file 1415-4757-GMB-46-3-s1-e20230103-s2.pdf]

Genetics and Molecular Biology
https://doi.org/10.1590/1678-4685-GMB-2023-0103

Supplementary Material to "Homocysteine and methylmalonic acid in Phenylketonuria patients"

Table S2 - Individual phenylalanine, methionine, B12, homocysteine and methylmalonic acid levels in patients before, after and pregnancy.

**METABOLITE**	**PT1 (N=13)**			**PT2 (N=32)**			**PT3 (N=20)**			**MEAN +/-SD**		
	BP (n=4)	P (n=9)	AP (n=0)	BP (n=0)	P (n=29)	AP (n=3)	BP (n=0)	P (n=19)	AP (n=1)	BP (n=4)	P (n=57)	AP (n=4)
**Age (yr)**	18.7	20	-	-	25	25.3	-	20.5	21	18.5 (0.5)	22.7 (2.3)	24.3
**Phe (µmol/L)**	1241	356	-	-	355	814	-	314	1073	1241 (409)	342 (293)	879 (343)
**Met (µmol/L)**	19.1	24.8	-	-	30.8	22.9	-	30.1	18.3	19.1 (6.31)	29.6 (10.3)	21.8 (2.4)
**tHcy (µmol/L)**	22.7	8.8	-	-	8.5	7.0	-	5,7	6.2	22.7 (11.3)	7.6 (3.9)	6.8 (1.7)
**MMA (µmol/L)**	0.20	0.22	-	-	0.17	0.19	-	0.23	0.42	0.21 (0.03)	0.20 (0.06)	0.25 (0.12)

BP: Before Pregnancy; AP: After Pregnancy; P: Pregnancy. Reference values: Phe: <360 µmol/L; Met: 16-34 µmol/L; tHcy: 5-15µmol/L; MMA: 0.1-0.5 µmol/L.
